# Resistance to Plazomicin: An Analysis of the Evidence from In Vitro Antimicrobial Susceptibility Studies

**DOI:** 10.3390/antibiotics15060559

**Published:** 2026-05-30

**Authors:** George Fanariotis, Panagiota Poziou, Laura T. Romanos, Iva D. Tzvetanova, Matthew E. Falagas

**Affiliations:** 1 Alfa Institute of Biomedical Sciences (AIBS), 9 Neapoleos Street, 15123 Athens, Greece; 2 School of Medicine, European University Cyprus, 2404 Nicosia, Cyprus; 3 Department of Medicine, Tufts University School of Medicine, Boston, MA 02111, USA

**Keywords:** ACHN-490, plazomicin, urinary-tract infections, complicated urinary-tract infections, Enterobacterales, *E. coli*, *Klebsiella*, CRE

## Abstract

**Introduction**: Plazomicin, a novel, semi-synthetic aminoglycoside designed to overcome most aminoglycoside-modifying enzymes (AMEs), represents a therapeutic alternative to traditional aminoglycosides for complicated urinary-tract infections (cUTIs). In this review, we sought to evaluate the available data on drug resistance. **Methods**: We performed a thorough search across four databases (PubMed, Embase, Scopus, and Web of Science) from their inception to 4 November 2025 to identify relevant studies. The published Clinical and Laboratory Standards Institute (CLSI) antimicrobial susceptibility breakpoints for Enterobacterales were applied. **Results**: Fifty-five studies, out of a total of 905 records originally identified, were eligible for data extraction and analysis, yielding antimicrobial susceptibility data for 80,159 clinical isolates. The overall resistance of consecutive Enterobacterales isolates to plazomicin was 0–9.4%, and specifically for *Escherichia coli* and *Klebsiella* spp. isolates were up to 4.7% and 15.2%, respectively. Among selected isolates with specific resistance mechanisms, the resistance of carbapenem-resistant Enterobacterales (CRE) was 1.8–67.6%, and specifically for carbapenem-resistant *E. coli* and *Klebsiella* spp. isolates, 0–10% and 0.3–90%, respectively. Among multi-drug-resistant (MDR) isolates, up to 24.6% of MDR *Klebsiella* spp. isolates and up to 15% of MDR *E. coli* isolates were resistant to plazomicin. Among non-fermenting Gram-negative bacteria, the MIC_90_ values were consistently high. **Conclusions**: The demonstrated high activity of plazomicin against consecutive Enterobacterales isolates and the considerable, yet variable, activity against selected resistant isolates suggest its consideration as a valuable option in the treatment of complicated urinary-tract infections.

## 1. Introduction

Urinary tract infections (UTIs) are encountered frequently in everyday clinical practice, both in the community and in hospitals. They are mainly caused by Gram-negative bacteria, usually Enterobacterales (*Escherichia coli*, *Proteus mirabilis*, *Klebsiella pneumoniae*, *Enterobacter* spp.) or, less frequently, by lactose non-fermenting bacteria (such as *Pseudomonas aeruginosa*) and Gram-positive bacteria (mainly *Staphylococcus* spp. and *Enterococcus* spp.). Rarely, UTIs are caused by fungi, primarily *Candida albicans* but also with increasing frequency other *Candida* spp. [1,2]. In the past, urine was thought to be sterile, a claim later rejected [3]. The presence of microorganisms in urine without symptoms (asymptomatic bacteriuria) is not an infection and should not be treated as one, except in special cases, such as pregnant women [2].

The predisposing factors for UTIs include the immunocompromised state, geriatric or frail patients, anatomic or functional abnormalities of the urinary tract, neuro-urological patients, female sex (including pregnancy and pelvic organ prolapse), antibiotic use in the past, indwelling urinary catheters, resistant organisms, stones, obstruction, recent instrumentation, post-void residual volume, benign prostatic obstruction and chronic bacterial prostatitis. Male sex alone is not considered a risk factor in the new classification due to a lack of supporting evidence [4]. The presence of a predisposing factor indicates that an infection is complicated. Τhe pathogenesis of a UTI starts with a fecal-perineal spread of a pathogen (the source) that contaminates the urethra and colonizes the bladder, leading to an ascending infection (the path) affecting the lower or upper urinary system, with the most serious outcome being bacteremia if the uropathogen crosses the kidney epithelial barrier [2].

Although UTIs rank as the second most common cause of infection leading to a prescribed antibiotic regimen (after respiratory tract infections), the symptoms and the commonly used diagnostic tools (urinalysis and urine culture) vary in sensitivity and specificity, while urine culture requires considerable time [1]. In addition, the treatment of UTIs becomes challenging in the context of systemic UTIs and the rising prevalence of aminoglycoside resistance [5]. Plazomicin, a new semi-synthetic aminoglycoside, rearms the arsenal against antibiotic resistance. It is a neoglycoside, or a “next generation”, semi-synthetic aminoglycoside [6]. Like other agents in this antibiotic class, it is administered once daily, as this dosing regimen for aminoglycosides reduces the risk of toxicity (nephrotoxicity and ototoxicity) [7,8].

Aminoglycosides exert their bactericidal effects by blocking bacterial protein synthesis. Their mechanism of action involves binding with high affinity to the A-site of the 16S rRNA, which is a component of the 30S ribosomal subunit [9]. The A-site serves as the aminoacyl-tRNA recognition site during translation [10]. The interaction between aminoglycosides and A-site causes the incorporation of near- or non-cognate tRNAs into the ribosome, and eventually, the production of aberrant protein chains. The accumulation of these defective proteins in the cytosol precipitates intracellular stress and ultimately induces a bactericidal effect [11].

Plazomicin utilizes the same mechanism of action as other clinically relevant aminoglycosides, such as gentamicin, tobramycin, and amikacin. However, it demonstrates a superior bactericidal profile against drug-resistant bacteria thanks to its unique structural qualities. Plazomicin is synthesized during an eight-step process from sisomicin, an analog of gentamicin 1a [12]. Key synthetic modifications provide resistance to isolates harboring inactivating enzymes known as aminoglycoside-modifying enzymes (AMEs). AMEs chemically modify aminoglycosides by N-acetylation (aminoglycoside acetyltransferases [AAC]), O-phosphorylation (aminoglycoside phosphotransferases [APH]), and adenylylation (aminoglycoside nucleotidyltransferases [ANT]). Thus, they compromise the aminoglycosides’ ability to bind appropriately to the A-site of the ribosome and diminish their bactericidal potency.

However, specific structural features of plazomicin overcome this limitation. Notably, two major synthetic modifications include the incorporation of a hydroxy-aminobutyric acid (HABA) group at the N-1 position and the addition of a hydroxyethyl substituent at the N-6 position. The N-1 substitution with HABA shields against AAC(3′), APH(2′), and ANT(2′), which are commonly produced AMEs in the Enterobacterales family. In addition, the presence of an unsaturated hydroxyethyl group at the 6′ position, a unique characteristic of plazomicin, confers resistance to the action of AAC(6′). Lastly, the lack of hydroxyl groups at the 3′ and 4′ positions protects against other inactivating enzymes such as ANT(4′) and APH(3′) [13].

Overall, these features outline the spectrum of activity against bacterial pathogens and the subsequent therapeutic indications. Plazomicin has been approved by the Food and Drug Administration (FDA) for the treatment of complicated UTIs (cUTIs) in adult patients [14]. In particular, it is expected to have a special role in cUTIs caused by drug-resistant pathogens harboring AME genes [15]. In this subgroup of infections, the conventional aminoglycosides often show limited efficacy [16]. The development of new antimicrobial agents can potentially provide valuable alternatives to traditional agents. In this setting, original data from in vitro and clinical studies, regarding the efficacy profiles and resistance trends of the novel antibiotics, are important to closely monitor and update their role in the current standard of care.

In this context, we sought evidence on bacterial resistance to this novel aminoglycoside. This review aims to identify potential weaknesses in the antimicrobial activity of plazomicin to aid physicians in its appropriate use.

## 2. Methods

### 2.1. Sources, Search Strategy, and Eligibility Criteria

We conducted a thorough search in four databases (Embase, PubMed, Scopus, Web of Science) from their inception to 4 November 2025. The detailed search strings are available in Supplementary File S2. It included words such as “plazomicin”, “antibiotic resistance”, and “minimum inhibitory concentration”. The inclusion criteria were as follows: (a) the terms plazomicin or “achn-490” included in the title/abstract/keywords, and (b) the study reported minimum inhibitory concentration (MIC). The exclusion criteria were (a) non-primary research articles; (b) case reports; (c) conference abstracts; (d) studies evaluating <10 total isolates for plazomicin susceptibility testing; (e) articles using isolates from animal sources; (f) primary research articles that did not contain relevant data for this review.

### 2.2. Screening of Studies, Data Extraction, and Tabulation

The automatic deduplication feature of the Rayyan tool was utilized. Two independent reviewers (G.F. and L.T.R.) screened the articles using their full text. A senior reviewer (M.E.F.) resolved any disagreements that arose during the screening. The data were extracted and tabulated by three independent reviewers (G.F., P.P., and L.T.R.). Data on bacterial species and their respective MIC range, MIC_50_, and MIC_90_ were reported as well as the number and proportion of resistant isolates to plazomicin according to Clinical and Laboratory Standards Institute (CLSI).

### 2.3. Breakpoints of Susceptibility Testing

At the time of writing, only the CLSI has published breakpoints for plazomicin resistance. CLSI first introduced susceptibility breakpoints for plazomicin in their CLSI M100-Ed33 version, published in 2023. These breakpoint cut-off values remain current, with CLSI M100-Ed36 being the latest available version, published in 2026. All data were retrospectively standardized using the CLSI M100-Ed36 guidelines. According to CLSI, isolates with MICs less than or equal to 2 mg/L are considered susceptible. Isolates with MIC values equal to 4 mg/L are considered intermediately resistant, while those with MIC values greater than or equal to 8 mg/L are considered resistant. It should be noted that these breakpoints do not apply to the Salmonella and Shigella species or the family of Morganellaceae, which includes the genera Morganella, Proteus, and Providencia. In instances where the proportions of resistant isolates to plazomicin were not reported by the authors of the studies included in our analysis, the above breakpoints were used to calculate them, based on the reported distribution of isolates per MIC, when possible. Given the lack of published in vitro susceptibility breakpoints for the Morganellaceae family and for Salmonella and Shigella species to plazomicin, we did not calculate the proportions of resistance among these isolates. We presented relevant resistance proportions for such isolates only when they were provided in the original records. Additionally, if the total number of Enterobacterales included isolates from the Morganellaceae family and excluding these isolates was not feasible during the secondary analysis, no relevant calculations were performed. 

### 2.4. Risk of Bias Assessment

We evaluated the risk of bias using a tool that was specifically developed for in vitro antimicrobial susceptibility studies [17]. Each record was evaluated across six domains for low, moderate, or high risk of bias. Based on the total scoring in each domain, each study was assigned a risk-of-bias rating of low, moderate, or high.

## 3. Results

### 3.1. Risk of Bias Assessment

The risk of bias evaluation for each study is available in a dedicated table in Supplementary File S1. 16 out of 55 studies (29%) were classified as having moderate risk of bias while the remainder were considered to have a high risk of bias. 

### 3.2. Selection of Relevant Articles

**Figure 1:**
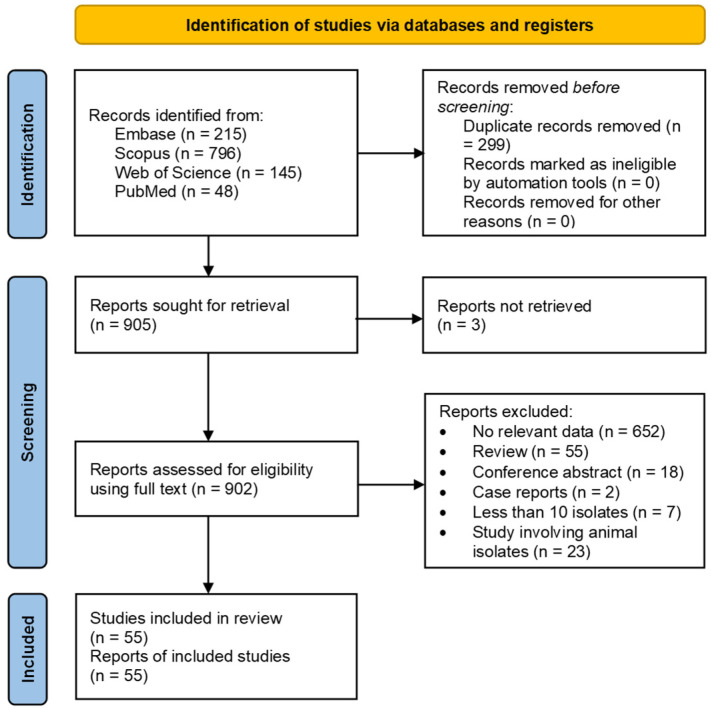
Flow diagram for the evaluation, selection, and inclusion of relevant articles.

The “Preferred Reporting Items for Systematic Reviews and Meta-Analyses” (PRISMA) flow diagram, shown in Figure 1, illustrates the search strategy and selection process used in this review. The initial search across the four databases yielded 1204 records. After removing duplicates, 905 records were sought for retrieval; 902 were successfully retrieved and assessed for eligibility. After applying the exclusion criteria, 55 articles were deemed eligible for inclusion in the review.

### 3.3. Main Findings

The 55 eligible articles that were included in our analysis yielded in vitro susceptibility data for a total of 80,159 clinical isolates. In Table 1, we present the relevant MIC ranges, MIC_50_, and MIC_90_ values, along with resistance data for the different isolate populations in each study after the application of the appropriate CLSI breakpoints. Consecutive isolates are listed exclusively in Table 1. The majority of the studies tested the effectiveness of plazomicin against members of the Enterobacterales family [18,19,20,21,22,23,24,25,26,27,28]. A subset of the articles assessed plazomicin’s effect against the Gram-negative, non-fermenting bacteria, *P. aeruginosa* and *Acinetobacter baumannii* [18,21,22,25,28,29]. A minority of the studies provided susceptibility data for Gram-positive cocci [18,21,22,28,30], whereas a single study evaluated the activity of plazomicin against *Mycobacterium abscessus* [31].

Regarding the consecutive isolates in Table 1, resistance frequency measures were presented or could be calculated after applying the latest CLSI breakpoints in six studies. Among these studies, the resistance of pooled Enterobacterales isolates ranged from 0% to 9.4% [21,22,23,24,25,27]. The resistance of *E. coli* to plazomicin ranged from 0.1% to 4.7% in seven studies. The intermediate resistance of *E. coli* isolates ranged from 0.4% to 7.7% in these seven studies [18,20,21,22,23,24,28]. Among the *Klebsiella* spp. isolates presented in seven studies, 0.1% to 15.2% were resistant to plazomicin [18,20,21,22,23,24,28]. The resistance of *Enterobacter* spp. isolates ranged from 0% to 2.9% across six studies [18,19,21,22,23,28].

In Table 2, we list studies reporting in vitro susceptibility data on selected clinical isolates. The authors of the relevant studies selected and tested clinical isolates based on phenotypic or genotypic characteristics conferring resistance to different antimicrobial agents [21,22,24,26,27,28,29,30,31,32,33,34,35,36,37,38,39,40,41,42,43,44,45,46,47,48,49,50,51,52,53,54,55,56,57,58,59,60,61,62,63,64,65,66,67,68,69,70,71,72].

Ten studies included data on the resistance of carbapenem-resistant Enterobacterales (CRE) to plazomicin, of which two reported non-susceptibility data rather than resistance proportions. In nine of these studies, the non-susceptibility ranged from 1.8% to 28.3%, whereas in the last one, 67.6% and 1% of the isolates were characterized as resistant and intermediate-resistant, respectively [21,22,24,27,41,43,49,54,64,70]. The resistance of carbapenem-resistant *Klebsiella* spp. isolates ranged from 0.3% to 35.6%, but a study from Bulgaria with 20 non-consecutive carbapenem-resistant and colistin-resistant isolates reported that 90% of the analyzed isolates were resistant to plazomicin [24,36,40,41,42,43,50,53,54,56]. The corresponding proportions for carbapenem-resistant *E. coli* and *Enterobacter* spp. isolates ranged from 0% to 10% [24,41,43,51] and from 3% to 40% [41,43,54], respectively. A subset of the studies evaluated the activity of plazomicin against multi-drug resistant (MDR) isolates, including both MDR Enterobacterales and MDR non-fermenting bacteria. In particular, 0% to 24.6% of the MDR *Klebsiella* spp. isolates were resistant, and 0% to 31.3% were intermediate-resistant to plazomicin [33,38,48,55,59,60,67,71]. In one of these studies, a resistance proportion of 53.8% is reported for *K. pneumoniae* complex isolates, excluding *K. pneumoniae* isolates [55]. The resistance of MDR *E. coli* isolates ranged from 0% to 15% with the intermediate resistance ranging from 0.3% to 17.2% [33,48,55,59,60,67], while the resistance of MDR *Enterobacter* spp. isolates was between 0% and 14.3% [33,48,55,60,67].

## 4. Discussion

Although 55 studies were eligible for data curation, this number of records does not ensure necessarily comprehensive representation of all isolate populations included in the analysis. Risk of bias was evaluated with a recently published risk-of-bias evaluation tool [17]. Only 16 out of 55 studies (29%) were classified as having a moderate risk of bias while the remainder were considered high risk. The increased number of studies characterized by high risk of bias, represents a substantial but not unexpected result which was primarily driven by mostly elevated scores in Domains 3 (Preparation bias) and 4 (Measurement/Observer bias) of the risk-of-bias evaluation tool. Issues pertinent to these domains, such as the absence of replicate experiments, or insufficient reporting regarding standard methodological procedures in antimicrobial susceptibility testing, classifies the relevant study as high risk of bias in the corresponding domain, and consequently as high risk of bias overall.

The thorough assessment of the susceptibility-related data presented in both tables yields important insights into the implications of the antimicrobial spectrum of plazomicin. The overall resistance of the drug against consecutive Enterobacterales isolates was up to 9.4% [21,22,23,24,25,27]. Consecutive isolates of different species within the Enterobacterales family demonstrated mostly low resistance, with the lowest resistance percentages observed among *E. coli* isolates, ranging from 0.1% to 4.7%, while slightly higher resistance percentages were observed among *Klebsiella* spp., ranging from 0.1% to 15.2% [18,20,21,22,23,24,28]. If an explanation were sought, this difference could likely be attributed to the higher proportion of antimicrobial-resistant *Klebsiella* spp. compared to *E. coli* isolates according to current epidemiological trends for these bacteria [73].

These observations provide an ideal starting point for analyzing the role of plazomicin in the treatment of infections caused by drug-resistant Enterobacterales strains. The results of this study on the resistance of plazomicin against selected CRE isolates were heterogeneous, ranging from 1.8% to 67.6% [21,22,24,27,41,43,49,54,64,70]. Smaller heterogeneity was observed among reported resistance percentages for MDR Enterobacterales isolates, ranging from 0% to 24.6% and from 0% to 15% for MDR *Klebsiella* spp. and MDR *E. coli*, respectively [33,38,48,55,59,60,67,71]. The variability in resistance results among the analyzed studies was multifactorial. Studies reporting higher MIC_90_ values and higher resistance proportions tended to include Morganellaceae in the resistance calculations and may have underestimated the true effectiveness of plazomicin against these pathogens. Beyond the consequences of including Morganellaceae in statistical analyses, further consideration should be given to the differences in resistance profiles and mechanisms among the selected isolates across studies. Compared populations of selected isolates across studies may harbor additional resistance mechanisms beyond those used as classification markers for initial analysis. The presence and lack of characterization of such resistance mechanisms could adversely affect the true resistance of plazomicin against selected isolates.

The effectiveness of plazomicin is completely compromised in the presence of genes encoding 16S rRNA methyltransferases, as this resistance mechanism confers resistance in 100% of isolates carrying these genes [22,44]. On the contrary, enzymatic resistance mechanisms, such as the presence of extended-spectrum β-lactamases (ESBLs) or carbapenemases, were associated with lower resistance, given the observed resistance proportions of less than 10% in carbapenemase-positive *E. coli*, *Klebsiella*, and *Enterobacter* isolates [34,38,45,46,65].

Plazomicin has also been extensively tested against non-fermenting Gram-negative bacteria, including *P. aeruginosa* and *A. baumannii*. However, the effectiveness of the antibiotic against these pathogens was rather poor, as evidenced by the mostly high MIC_90_ values, ranging from 8 mg/L to 64 mg/L and from 4 to ≥128 mg/L, for *Pseudomonas* and *Acinetobacter*, respectively [21,22,25,28,29]. These results were anticipated and justified by the heavy reliance of these non-fermenters on non-enzymatic resistance mechanisms, such as efflux pumps and reduced membrane permeability [74]. Plazomicin lacks the tools necessary to evade these resistance mechanisms, as it was engineered to overcome resistance conferred by most AMEs [75].

Lastly, special attention should be paid to the family Morganellaceae within Enterobacterales. The consecutive isolates in this family demonstrated consistently higher MIC_90_ values, more than 4 mg/L in most cases, in comparison with the corresponding MIC_90_ values for other members of the Enterobacterales family [18,21,22,24,26,28]. These results, coupled with the fact that published susceptibility breakpoints for Enterobacterales are not applicable to the Morganellaceae family, according to the latest CLSI guidance, indicate the presence of an underlying intrinsic resistance mechanism that reduces the susceptibility of this bacterial family to plazomicin.

Although plazomicin retains activity against isolates producing a wide range of AMEs, it shows decreased potency when the modifying enzymes AAC(2′)-Ia and APH(2″)-IVa are expressed. Expression of AAC(2′)-Ia, which is primarily present in *Providencia stuartii* isolates and occasionally in *Acinetobacter* spp., mediates plazomicin acetylation. It increased the drug’s MIC by 16-fold in a relevant study [75]. Similarly, APH(2″)-IVa mediates the phosphorylation of the 2″-OH group of plazomicin, leading to a 4- to 8-fold increase in plazomicin MIC, as reported in the same study [75]. Plazomicin, as previously stated, does not retain activity when a 16S rRNA ribosomal methyltransferase is expressed, as it modifies the bacterial ribosome, the drug’s target, leading to significantly higher plazomicin MIC values [13,26,75,76]. Also, plazomicin is susceptible to altered expression of efflux pumps and porin channels, leading to reduced intracellular drug concentrations and reduced antibacterial efficacy. Finally, the drug’s ability to cross cytoplasmic membranes is energy dependent and is strongly influenced by anaerobic or acidic conditions. Therefore, plazomicin, like other aminoglycosides, demonstrates limited efficacy in acidic or anaerobic environments [10].

Plazomicin has been studied in a multicenter, randomized, double-blind, noninferiority trial involving adult patients (≥18 years) with documented cUTIs, including acute pyelonephritis [77]. Randomization was 1:1 to receive either intravenous plazomicin (15 mg/kg daily) or intravenous meropenem (1 g every 8 h), for 4 days followed by optional oral step-down therapy (usually levofloxacin) for a total treatment duration of 7–10 days. Composite cure was defined as clinical cure and microbiological eradication assessed on day 5 and during a test-of-cure visit 15 to 19 days later. 

Evaluation of the efficacy outcomes demonstrated that plazomicin met the criteria for noninferiority compared with meropenem across both prespecified primary endpoints. At day 5, the composite clinical and microbiologic cure was 88.0% in the plazomicin arm and 91.4% in the meropenem arm, yielding a between-group difference of −3.4 percentage points, which remained well within the established noninferiority margin of −15%. These findings suggest comparable early efficacy in symptom resolution and bacterial clearance. Results at the test-of-cure visit further reinforced noninferiority, with cure of 81.7% for plazomicin and 70.1% for meropenem, reflecting an absolute difference of 11.6 percentage points in favor of plazomicin. This trend indicates that plazomicin may sustain therapeutic benefit over time.

Secondary analyses supported these observations, as plazomicin achieved high microbiologic eradication proportions, exceeding 98% at earlier assessments and remaining above 89% at follow-up [15]. These outcomes were particularly notable among infections caused by resistant organisms, including ESBL-producing pathogens.

Further subgroup analyses demonstrated superior microbiologic activity of plazomicin against select resistant strains. Among patients infected with ESBL-producing Enterobacterales, eradication at the test-of-cure visit occurred in 82.4% of those receiving plazomicin, compared with 75.0% in the meropenem group. Similarly, in infections involving clinical isolates resistant to other aminoglycosides, plazomicin achieved an eradication proportion of 78.8%, exceeding the 68.6% observed with meropenem. These results underscore plazomicin’s enhanced effectiveness against MDR pathogens and highlight its potential role in addressing challenging resistant infections.

Common adverse events are those that occur in 1% or more of patients. In one Phase 3 trial (NCT02486627) that included patients receiving plazomicin as monotherapy, the most common adverse event was decreased renal function. It occurred in 3.6% of the 303 patients and in 1.3% of the 301 patients in the plazomicin and the meropenem group, respectively. It was followed by diarrhea and hypertension, which both occurred in 2.3% of patients in the plazomicin group and in 1.7% and 2.3% of patients in the meropenem group, respectively. In the plazomicin group, headache, nausea, and vomiting occurred in 1.3% of patients each, while 3%, 1.3% and 1% of patients in the meropenem group experienced these symptoms, respectively. Hypotension was experienced by 1% and 0.7% of patients in the plazomicin and meropenem groups, respectively [77]. The adverse reactions observed in a Phase 2 trial (NCT01096849) were similar to those observed in this trial [78].

Additional adverse reactions reported in both aforementioned trials in more than one patient in the plazomicin groups were gastrointestinal disorders (constipation, gastritis), increased alanine aminotransferase, hypokalemia, dizziness, hematuria, and dyspnea [77,78]. Patients may also experience decreased renal function and alterations in blood pressure [79].

The FDA product information leaflet of the drug lists some warnings and precautions. Nephrotoxicity is a particular concern and has been reported more frequently than with other new antimicrobial agents [80]. One case of reversible hypoacusis and one case of tinnitus and reversible vertigo were recorded during clinical trials NCT02486627 and NCT01096849, respectively [14]. Plazomicin is a member of the aminoglycoside class, such complications are to be expected [8].

Beyond the FDA-approved indication, the CARE clinical trial, NCT01970371, attempted to study the role of plazomicin in the treatment of systemic infections caused by CRE by comparing plazomicin to colistin for the treatment of confirmed CRE infections [81]. This trial was terminated prematurely due to poor patient enrollment and, therefore, failed to provide meaningful evidence of its efficacy that would establish plazomicin as a better option for CRE infections over colistin. Nevertheless, the results for the CARE trial on 37 patients with confirmed CRE infections had been promising [82]. The primary end point, being a composite of death from any cause or significant disease-related complications, was observed in 24% of patients under plazomicin and adjunctive meropenem or tigecycline, compared with 50% of patients who received colistin [82]. This limited data had provided the ground for the potential use of plazomicin in the treatment of severe systemic infections beyond its current role in the treatment of targeted infections of the urinary tract. Ten studies included in this review provide susceptibility data on CRE isolates, demonstrating notable variability in resistance to plazomicin [21,22,24,27,41,43,49,54,64,70]. The factors contributing to the observed resistance heterogeneity should be identified. Until then, the geographical background of each study which reflects the local epidemiology seems to be an apparent contributor to this phenomenon. In this sense, additional clinical and in vitro antimicrobial susceptibility data are warranted to elucidate plazomicin’s role in the modern treatment landscape of systemic infections.

Certain limitations of the current study should be addressed. First, its protocol was not registered in a relevant, publicly accessible repository. Second, the lack of a risk of bias assessment tool prevented us from conducting an analogous evaluation of the records included in the study. Third, the extraction of the number and, subsequently, of the proportion of resistant isolates from the reported MIC distributions of the analyzed pathogens was required in some studies, as part of a necessary secondary analysis when these data were not readily available. Lastly, certain studies did not exclude but used MIC data of isolates belonging to the Morganellaceae family in the calculation of resistance proportions for the Enterobacterales family, despite the non-applicability of the published susceptibility breakpoints for these specific pathogens.

## 5. Conclusions

Plazomicin, a novel, semi-synthetic aminoglycoside designed to evade most aminoglycoside-modifying enzymes, represents a promising option for the treatment of complicated urinary-tract infections. The present evaluation of published in vitro susceptibility data provides evidence of low resistance among Enterobacterales, with retained yet variable activity against selected carbapenem-resistant and MDR Enterobacterales isolates. In contrast, plazomicin remains ineffective against non-fermenting Gram-negative bacteria, such as *Pseudomonas* spp. and *Acinetobacter* spp. Consequently, plazomicin should be considered a clinically useful alternative to traditional aminoglycosides for the treatment of patients with complicated urinary-tract infections. Given the variability of resistance among selected isolates, clinicians should integrate in vitro antimicrobial susceptibility testing results into decisions regarding the use of plazomicin, when available, to optimize clinical outcomes.

## Figures and Tables

**Table 1:** Resistance of consecutive isolates to plazomicin.

Author *	Year	Isolates	N	MIC Range/Value (mg/L)	MIC_50_ (mg/L)	MIC_90_ (mg/L)	Resistance n (% **)
Huang [31]	2025	*M. abscessus*	39	>16	>16	>16	NA
Zhanel [28]	2025	*E. coli*	11,932	≤0.03–>64	0.5	1	12 (0.1), I: 72 (0.6)
		*P. aeruginosa*	5961	≤0.12–64	4	16	NA
		*K. pneumoniae*	3973	≤0.12–>64	0.25	0.5	4 (0.1), I: 4 (0.1)
		*E. cloacae*	1659	≤0.12–64	0.5	0.5	3 (0.2), I: 3 (0.2)
		*P. mirabilis*	957	0.25–32	4	8	NA
		*K. oxytoca*	1060	≤0.12–2	0.5	1	0 (0)
		*S. marcescens*	1003	≤0.12–8	1	2	8 (0.8), I: 21 (2.1)
		*K. aerogenes*	437	≤0.12–2	0.5	1	0 (0)
		*C. freundii*	260	≤0.12–2	0.5	1	0 (0)
		*A. baumannii*	274	0.25–>64	1	4	NA
Camargo [29]	2023	*P. aeruginosa*	119	0.125–>64	8	16	NA
Sader [27]	2023	Enterobacterales	9809	0.06–≥128	0.5	1	82 (0.8), Ι: 267 (2.7) ^a^
Blanchard [26]	2022	Enterobacterales	598	0.125–≥256	0.5	4	NA
Gysin [25]	2021	Enterobacterales	25	0.125–2	NA	NA	0 (0)
Gysin	2021	*P. aeruginosa*	32	0.5–8	NA	NA	NA
Ince [24]	2021	Enterobacterales	180	NA	0.5	2	17 (9.4)
		*K. pneumoniae*	105	NA	0.5	>256	16 (15.2)
		*E. coli*	75	NA	0.5	1	1 (1.3)
Gür [23]	2020	Enterobacterales	714	NA	0.25	2	60 (8.4)
		*E. coli*	320	NA	0.25	1	15 (4.7)
		*Klebsiella* spp.	294	NA	0.25	128	34 (11.6)
		*Enterobacter* spp.	69	NA	0.25	0.5	1 (1.4)
		*S. marcescens*	20	NA	0.5	8	5 (20)
		*Citrobacter* spp.	11	NA	0.25	1	0 (0)
Zhanel [32]	2019	MSSA	7146	≤0.12–16	0.5	1	NA
		MRSA	1963	≤0.12–64	0.5	1	NA
		Community-acquired MRSA	640	≤0.12–4	0.5	1	NA
		Hospital-acquired MRSA	1237	≤0.12–64	1	1	NA
		Methicillin-S *S. epidermidis*	468	≤0.12–0.5	≤0.12	0.25	NA
		Methicillin-R *S. epidermidis*	103	≤0.12–4	0.25	0.5	NA
		*S. pneumoniae*	2626	≤0.12–64	16	32	NA
		*S. agalactiae*	689	16–>64	64	>64	NA
		*S. pyogenes*	642	4–64	16	32	NA
		*E. faecalis*	1255	≤1–>512	4	4	NA
		*E. faecium*	481	1–>64	8	16	NA
		VRE	106	1–32	8	16	NA
		*S. maltophilia*	667	≤1–32	32	>32	NA
Castanheira [21]	2018	Enterobacterales	4362	≤0.06–>128	0.5	2	6 (0.2), I: 14 (0.4) ^b^
		*K. pneumoniae*	1506	≤0.06–>128	0.25	0.5	2 (0.1), I: 1 (0.06)
		*E. coli*	1346	≤0.06–>128	0.5	1	2 (0.1), I: 6 (0.4)
		*K. oxytoca*	359	≤0.06–>128	0.5	0.5	2 (0.6), I: 1 (0.3)
		*C. freundii* spp. complex	159	0.12–4	0.5	1	0 (0)
		*Providencia* spp.	158	0.12–64	2	4	NA
		*C. koseri*	145	≤0.06–4	0.25	0.5	1 (0.7)
		*P. mirabilis*	124	0.25–8	2	4	NA
		*E. aerogenes*	120	≤0.06–>4	0.5	1	I: 1 (0.8)
		*M. morganii*	118	0.5–64	2	4	NA
		*P. vulgaris* group	116	0.5–16	2	4	NA
		*S. marcescens*	107	0.12–4	1	2	I: 3 (2.8)
		*E. cloacae* spp.	104	0.12–>2	0.5	0.5	0 (0)
		*P. aeruginosa*	103	0.12–>128	4	16	NA
		*Acinetobacter* spp.	95	≤0.06–>128	2	16	NA
		CoNS	72	≤0.06–0.5	0.12	0.5	NA
		*S. aureus*	69	0.25–2	0.5	0.5	NA
		MRSA	30	0.25–2	0.5	0.5	NA
		*S. pneumoniae*	66	16–64	32	64	NA
		*Enterococcus* spp.	58	1–128	16	64	NA
Castanheira [22]	2018’	Enterobacterales	4217	≤0.06–>128	0.5	2	63 (1.7), I: 7 (0.2) ^c^
		*K. pneumoniae*	1429	≤0.06–>128	0.25	0.5	60 (4.2)
		*E. coli*	1399	0.12–16	0.5	1	2 (0.1), I: 6 (0.4)
		*K. oxytoca*	317	0.12–2	0.5	0.5	0 (0)
		*C. koseri*	145	≤0.06–1	0.25	0.5	0 (0)
		*C. freundii*	131	0.12–4	0.5	1	0 (0), I: 1 (0.8)
		*M. morganii*	131	0.25–16	2	4	NA
		*E. aerogenes*	129	≤0.06–2	0.5	1	0 (0)
		*E. cloacae*	119	0.12–1	0.25	0.5	0 (0)
		*P. mirabilis*	119	0.5–>128	2	4	NA
		*S. marcescens*	105	0.25–8	1	1	1 (1)
		*P. vulgaris* group	109	0.25–8	1	2	NA
		*Providencia* spp.	84	0.5–>128	2	8	NA
		*P. aeruginosa*	102	0.12–64	4	8	NA
		*Acinetobacter* spp.	99	≤0.06–128	8	>128	NA
		*S. aureus*	69	0.12–2	0.5	1	NA
		MRSA	20	0.25–1	0.5	0.5	NA
		CoNS	67	≤0.06–1	0.12	0.25	NA
		*S. pneumoniae*	67	8–64	32	64	NA
		*Enterococcus* spp.	59	0.12–128	32	128	NA
Walkty [19]	2014	*E. aerogenes*	55	≤0.12–2	0.25	0.5	NA
Tenover [30]	2011	MRSA	493	≤0.12–8	1	2	NA
Aggen ^d^ [18]	2010	*Citrobacter* spp.	24	≤0.25–4	1	2	0 (0), I: 2 (8.3)
		Aminoglycoside-S	15	1–4	2	4	I: 2 (13.3)
		Aminoglycoside-R	9	≤0.25–1	0.5	1	0 (0)
		*E. coli*	39	0.5–16	1	4	1 (2.6), I: 3 (7.7)
		Aminoglycoside-S	15	0.5–4	2	4	I: 3 (20)
		Aminoglycoside-R	24	0.5–16	1	2	1 (4.2)
		*Enterobacter* spp.	35	≤0.25–8	0.5	2	1 (2.9), I: 1 (2.9)
		Aminoglycoside-S	15	≤0.25–4	0.5	2	I: 1 (6.7)
		Aminoglycoside-R	20	0.25–8	0.5	1	1 (5)
		*Klebsiella* spp.	60	≤0.25–8	0.5	1	1 (1.7)
		Aminoglycoside-S	15	≤0.25–1	0.5	1	0 (0)
		Aminoglycoside-R	45	≤0.25–8	0.5	1	1 (2.2)
		*P. mirabilis*	23	1–16	4	8	NA
		Aminoglycoside-S	7	2–16	4	16	NA
		Aminoglycoside-R	16	1–16	4	8	NA
		*Salmonella* spp., *Shigella* spp.	14	0.5–8	2	8	NA
		Aminoglycoside-S	13	0.5–8	2	8	NA
		Aminoglycoside-R	1	0.5	NA	NA	NA
		*Serratia* spp.	28	0.5–4	2	2	I: 2 (7.1)
		Aminoglycoside-S	20	1–4	2	2	I: 1 (5)
		Aminoglycoside-R	8	0.5–4	1	4	I: 1 (12.5)
		*Staphylococcus* spp.	59	≤0.25–4	0.5	2	NA
		Aminoglycoside-S	10	≤0.25–2	0.5	1	NA
		Aminoglycoside-R	49	≤0.25–4	1	2	NA
		*Acinetobacter* spp.	82	0.5–>64	8	32	NA
		Aminoglycoside-S	15	0.5–4	2	2	NA
		Aminoglycoside-R	67	1–>64	16	32	NA
		*P. aeruginosa*	66	0.5–>64	8	32	NA
		Aminoglycoside-S	15	2–16	4	4	NA
		Aminoglycoside-R	51	0.5–>64	8	64	NA
		*Proteae* indole+	31	1–16	4	8	NA
		Aminoglycoside-S	23	1–16	2	4	NA
		Aminoglycoside-R	8	4–16	8	NA	NA
Landman [20]	2010	*E. coli*	3050	≤0.06–>8	0.5	1	NA
		*K. pneumoniae*	1155	0.12–>8	0.5	1	NA

* Studies are presented in descending chronological order (and alphabetical order within a year). Studies by the same first author, published within the same calendar year are ordered in descending chronological order by month of publication. ** All percentage figures were rounded to one decimal place, except the values < 0.1 that were rounded to two decimal places. Notes: ^a^ Isolates from the family of Morganellaceae were included in statistical analysis for resistance calculation, ^b^ Resistance calculated after secondary analysis with exclusion of Morganellaceae. Resistance data refer to a net population of 3846 Enterobacterales isolates. ^c^ Resistance was calculated after secondary analysis with exclusion of Morganellaceae. Resistance data refer to a net population of 3774 Enterobacterales isolates, ^d^ Susceptibility or resistance to aminoglycosides status was reported according to resistance status against amikacin, gentamicin, and tobramycin. Aminoglycoside-susceptible isolates demonstrated amikacin MICs of ≤16 mg/L and gentamicin and tobramycin MICs of ≤4 mg/L. Isolates with amikacin MICs of >16 mg/L, and/or gentamicin MICs of >4 mg/L and/or tobramycin MICs of >4 mg/L were classified as aminoglycoside-resistant isolates. Abbreviations: *A. baumannii*, *Acinetobacter baumannii*; *C. freundii*, *Citrobacter freundii*; *C. koseri*, *Citrobacter koseri*; CoNS, coagulase-negative *Staphylococci*; *E. aerogenes*, *Enterobacter aerogenes*; *E. cloacae*, *Enterobacter cloacae*; *E. coli*, *Escherichia coli*; *E. faecalis*, *Enterococcus faecalis*; *E. faecium*, *Enterococcus faecium*; I, intermediate; indole+, indole positive; *K. aerogenes*, *Klebsiella aerogenes*; *K. oxytoca*, *Klebsiella oxytoca*; *K. pneumoniae*, *Klebsiella pneumoniae*; MIC, minimum inhibitory concentration; MIC_50_, minimum inhibitory concentration 50%; MIC_90_, minimum inhibitory concentration 90%; *M. abscessus*, *Mycobacterium abscessus*; *M. morganii*, *Morganella morganii*; MRSA, methicillin-resistant *Staphylococcus aureus*; MSSA, methicillin-sensitive *staphylococcus aureus*; NA, non-applicable; *P. aeruginosa*, *Pseudomonas aeruginosa*; *P. mirabilis*, *Proteus mirabilis*; *P. vulgaris*, *Proteus* vulgaris; R, resistant; S, susceptible; *S. aureus*, *Staphylococcus aureus*; *S. epidermidis*, *Staphylococcus epidermidis*; *S. marcescens*, *Serratia marcescens*; *S. maltophilia*, *Stenotrophomonas maltophilia*; *S. agalactiae*, *Streptococcus agalactiae*; *S. pneumoniae*, *Streptococcus pneumoniae*; *S. pyogenes*, *Streptococcus pyogenes*; VRE, vancomycin-resistant *enterococcus*.

**Table 2:** Resistance of selected isolates to plazomicin.

Author *	Year	Isolates	N	MIC Range/Value (mg/L)	MIC_50_ (mg/L)	MIC_90_ (mg/L)	Resistance n (% **)
Zhanel [28]	2025	ESBL+ *E. coli*	1007	≤0.12–32	0.5	1	2 (0.2), I: 3 (0.3)
		ESBL+ *K. pneumoniae*	221	≤0.12–>64	0.25	0.5	2 (0.8), I: 2 (0.1)
Dahdouh [34]	2024	Carbapenemase+ *E. coli*	90	NA	1.5	2	7 (7.8)
Halim [35]	2024	PDR, XDR *A. baumannii* ^a,b^	21	6–>32	>32	>32	NA
Markovska [36]	2024	Carbapenem-R and colistin-R *K. pneumoniae*	20	NA	NA	NA	18 (90)
Markovska [37]	2024’	Carbapenemase+ Enterobacterales	64	≤0.25–>256	0.5	4	6 (9.4), I: 2 (3.1) ^c^
Sękowska [38]	2024	Carbapenemase+ *K. pneumoniae*	60	0.25–256	0.5	0.75	NS: 1 (1.7)
		MDR, ESBL+ *K. pneumoniae* ^d^	50	0.19–4	0.5	0.75	NS: 2 (4)
Słabisz [39]	2024	NDM *K. pneumoniae*	60	0.38–1	0.75	0.75	13 (22)
Cañada-García [46]	2023	Carbapenemase+ *K. pneumoniae*	85	0.125–8	0.5	0.1	1 (1.2), I: 1 (1.2)
Maraki [40]	2023	Carbapenem-R *K. pneumoniae*	110	NA	0.5	1.5	NS: 7 (6.4)
Sader ^c^ [27]	2023	Carbapenem-R Enterobacterales	117	0.06–≥128	0.25	1	6 (5.1), Ι: 1 (0.9)
		ESBL+	1011	0.06–≥128	0.5	1	9 (0.9), I: 2 (0.2)
		AME+	801	0.06–≥128	0.5	1	11 (1.4), I: 10 (1.3)
		Amikacin-NS	528	0.12–≥128	1	4	34 (6.4), I: 38 (7.2)
		MDR ^d^	844	0.06–≥128	0.25	1	21 (2.5), I: 23 (2.7)
		XDR ^b^	84	0.12–≥128	0.25	1	5 (6)
Teo [41]	2023	Carbapenem-R Enterobacterales	365	≤0.25–≥512	0.5	≥512	69 (18.9), I: 1 (0.3)
		*K. pneumoniae*	153	NA	1	≥512	NS: 56 (36.6)
		*E. coli*	93	NA	1	2	NS: 5 (5.4)
		*E. cloacae*	88	NA	0.5	2	NS: 8 (9.1)
Zhang [42]	2023	Carbapenem-R *K. pneumoniae*	375	≤0.5–≥8	NA	NA	1 (0.3), I: 2 (0.5)
Arca-Suárez [44]	2022	Enterobacterales ^e^	24	NA	>256	>256	24 (100)
Blanchard ^f^ [26]	2022	*K. pneumoniae*	17	0.25–>256	2	256	7 (41.2), I: 1 (5.9)
		*K. oxytoca*	7	0.25–2	0.5	2	0 (0)
		*K. aerogenes*	6	0.5–8	0.5	8	1 (16.7)
		*E. coli*	13	0.25–>256	4	256	5 (38.5), I: 2 (15.4)
		*E. cloacae*	10	0.25–0.5	0.25	0.5	0 (0)
		*C. freundii*	6	0.25–1	0.5	1	0 (0)
		*C. koseri*	3	0.25–2	0.25	2	0 (0)
		*M. morganii*	3	2–16	8	16	NA
		*P. mirabilis*	4	2–32	4	32	NA
		*P. vulgaris* group	5	2–4	4	4	NA
		*P. stuartii*	2	1–256	1	256	NA
		*S. marcescens*	4	1	1	1	0 (0)
Cañada-García [45]	2022	Carbapenemase+ *K. pneumoniae*	377	0.25–>256	1	2	23 (6.1), I: 2 (0.5)
		Carbapenemase+ *E. coli*	26	0.06–2	1	2	0 (0)
Gadallah [43]	2022	Carbapenem-R isolates	61	0.12–>128	1	16	NA
		Carbapenem-R Enterobacterales	45	0.12–>128	1	8	NS: 8 (17.8) ^c^
		*K. pneumoniae*	22	0.128–16	1	8	3 (13.6), I: 1 (4.5)
		*E. coli*	13	0.25–2	0.5	1	0 (0)
		*Enterobacter* spp.	5	0.5–16	1	16	2 (40)
		*K. oxytoca*	2	8–>128	8	>128	2 (100)
		*P. mirabilis*	2	0.5–1	0.5	1	NA
		*Citrobacter* spp.	1	2	NA	NA	0 (0)
		Carbapenem- R NLF isolates	16	0.5–>128	4	>128	NA
		*P. aeruginosa*	10	0.5–>128	4	>128	NA
		*Acinetobacter* spp.	5	2–64	8	64	NA
		*Stenotrophomonas* spp.	1	>128	NA	NA	NA
Gysin [33]	2022	MDR Enterobacterales	422	≤0.25–>256	1	8	49 (11.6), I: 20 (4.7) ^c^
		*E. coli*	242	0.5–>256	1	4	14 (5.8), I: 15 (6.2)
		*Klebsiella* spp.	134	≤0.25–>256	0.5	256	33 (24.6), I: 3 (2.2)
		*Enterobacter* spp.	33	0.5–256	0.5	1	1 (3)
		Other	13	0.5–8	1	4	1 (7.7), I: 2 (15.4)
		MDR *Acinetobacter* spp.	30	0.5–>256	4	>256	NA
		MDR *P. aeruginosa*	18	16–>256	>256	>256	NA
Abd-Elmonsef [47]	2021	Fluoroquinolone-R isolates ^g^	127	0.06–64	1	4	NA
		Fluoroquinolone-R Enterobacterales ^g^	98	0.06–4	1	2	4 (4) ^c^
		*K. pneumoniae*	53	0.12–4	1	2	I: 2 (3.8)
		*E. coli*	37	0.12–4	1	2	I: 1 (2.7)
		*Proteus* spp.	4	1–4	2	4	NA
		*Enterobacter* spp.	4	0.06–0.25	0.25	0.25	0 (0)
		Fluoroquinolone-R *P. aeruginosa* ^g^	17	0.25–32	2	16	NA
		Fluoroquinolone-R *Acinetobacter* spp. ^g^	12	2–64	4	32	NA
Albano [48]	2021	MDR Enterobacterales	296	0.125–>128	1	>128	41 (80) ^c^
		*E. coli*	94	0.5–>128	2	128	14 (14.9)
		*K. pneumoniae*	151	0.125–>128	0.5	>128	22 (15.6)
		*K. aerogenes*	3	0.5–1	0.5	1	0 (0)
		*E. cloacae*	14	0.25–>128	0.5	8	2 (14.3)
		*E. cloacae* complex	7	0.25–1	0.5	1	0 (0)
		*C. freundii*	4	0.25–0.5	0.25	1	0 (0)
		*C. freundii* complex	2	0.5–1	0.5	1	0 (0)
		*C. koseri*	4	0.25–0.5	0.5	0.5	0 (0)
Essam [49]	2021	Carbapenem-R Enterobacterales	102	0.25–>256	>256	>256	69 (67.6), I: 1 (1) ^c^
Huang [50]	2021	Carbapenem-R *K. pneumoniae*	158	≤0.125–64	0.25	0.5	1 (0.63)
Ince [24]	2021	Carbapenem-R isolates	60	NA	0.5	>256	17 (28.3)
		Carbapenem-R *K. pneumoniae*	45	NA	0.5	>256	16 (35.6)
		Carbapenem-R *E. coli*	15	NA	0.5	1	1 (6.7)
		ESBL+ isolates	60	NA	0.5	1	0 (0)
		ESBL+ *K. pneumoniae*	30	NA	0.25	0.5	0 (0)
		ESBL+ *E. coli*	30	NA	0.5	1	0 (0)
		ESBL– isolates	60	NA	0.5	1	0 (0)
		ESBL− *K. pneumoniae*	30	NA	0.25	0.5	0 (0)
		ESBL− *E. coli*	30	NA	0.5	1	0 (0)
Johnston [52]	2021	Cephalosporin-R *E. coli* ^h^	216	0.025–4	1	2	1 (0.5)
Johnston [51]	2021’	Carbapenem-R *E. coli*	343	0.12–>256	1	4	34 (10), I: 3 (1)
Maraki [53]	2021	Carbapenem-R *K. pneumoniae*	266	≤0.25–256	0.38	1.5	4 (1.5)
Clark [54]	2020	Carbapenem-R Enterobacterales	122	0.06–>32	0.5	1	NS: 2 (2)
		*Klebsiella* spp.	63	0.125–>32	0.5	0.5	1 (1)
		*Enterobacter* spp.	40	0.06–4	0.25	0.5	1 (3)
Fleischmann [55]	2020	MDR Enterobacterales	303	0.125–>128	1	128	NA
		*K. pneumoniae*	144	0.125–>128	0.5	128	17 (11.8)
		*E. coli*	93	0.5–>128	2	128	14 (15), I: 16 (17.2)
		*E. cloacae*	14	0.25–128	0.5	128	2 (14.3)
		*K. pneumoniae* complex	13	0.25–>128	8	>128	7 (53.8)
		*E. cloacae* complex	7	0.25–1	0.5	1	0 (0)
		*S. marcescens*	5	1–8	2	8	1 (20)
		*C. freundii*	4	0.25–0.5	0.25	0.5	0 (0)
		*C. freundii* complex	4	0.5–1	0.5	1	0 (0)
		*C. koseri*	4	0.25–0.5	0.5	0.5	0 (0)
		*K. aerogenes*	3	0.5–1	0.5	1	0 (0)
		*M. morganii*	2	2	2	2	NA
		*P. stuartii*	2	0.5–4	0.5	4	NA
		*C. sedlakii*	1	>128	NA	NA	1 (100)
		*K. oxytoca*	1	1	NA	NA	0 (0)
		*P. mirabilis*	1	4	NA	NA	NA
Jacobs [56]	2020	Carbapenem-R *K. pneumoniae*	697	≤0.12–>32	0.25	1	17 (2.4)
Galani [57]	2019	Carbapenem-NS *K. pneumoniae*	300	0.125–>256	0.5	4	26 (8.7), I: 13 (4.3)
		KPC	201	0.125–>256	1	4	19 (9.5), I: 11 (5.5)
		NDM	52	0.125–2	0.5	1	0 (0)
		VIM	21	0.125–>256	1	>256	6 (28.6)
		OXA-48	12	0.125–4	0.5	2	I: 1 (8.3)
		KPC, VIM	14	0.25–2	1	2	I: 1 (7.1)
Savov [58]	2019	MDR *A. baumannii*	28	2–>256	>256	>256	NA
Savov	2019	MDR *P. aeruginosa*	28	1.5–16	8	16	NA
Walkty [59]	2019	MDR *E. coli* ^d^	358	≤0.12–>64	0.5	1	1 (0.3), I: 1 (0.3)
		MDR *K. pneumoniae* ^d^	74	≤0.12–>64	0.25	0.5	1 (1.3), I: 1 (1.4)
		MDR *P. aeruginosa* ^d^	256	≤0.12–>64	8	64	NA
		Gentamicin-S *S. aureus*	3626	≤0.12–4	0.5	1	NA
		Gentamicin-NS *S. aureus*	69	0.25–16	1	1	NA
		Gentamicin-S *S. epidermidis*	240	≤0.12–0.5	≤0.12	0.25	NA
		Gentamicin-NS *S. epidermidis*	124	≤0.12–2	0.25	0.25	NA
Castanheira [21]	2018	Carbapenem-R Enterobacterales	97	≤0.06–>128	0.5	1	NA
Castanheira [22]	2018’	Carbapenem-R Enterobacterales	227	≤0.25–>128	0.25	128	NA
		16S rRNA methyltransferases gene+	60	128–>128	>128	>128	NA
Thwaites [60]	2018	MDR Enterobacterales	30	0.12–8	0.5	2	NA
		*E. coli*	10	0.5–4	1	4	NA
		*Klebsiella* spp.	8	0.25–8	0.5	NA	NA
		*Enterobacter* spp.	10	0.25–2	0.5	1	0 (0)
		*C. freundii*	2	0.12–0.5	NA	NA	0 (0)
Denervaud-Tendon [61]	2017	Colistin-R Enterobacterales (acquired, chromosomal resistance)	42	0.25–>128	0.25	1	4 (9.5)
		Colistin-R Enterobacterales (unknown mechanism)	24	0.12–>128	0.5	4	2 (8.3)
		Colistin-R Enterobacterales (acquired plasmid mcr-1	21	0.5–2	1	2	0 (0)
		Colistin-R Enterobacterales (intrinsic resistance)	8	1–4	NA	NA	0 (0)
López Díaz [63]	2017	MRSA	55	0.125–2	0.5	1	NA
Martins [62]	2017	Carbapenemase+ Enterobacterales	499	≤0.125–>64	0.5	64	NA
Zhang [64]	2017	Carbapenem-R Enterobacterales	110	0.25–≥32	0.5	1	2 (1.8), I: 2 (1.8)
Rodríguez-Aviala [65]	2015	Carbapenemase+ Enterobacterales	164	0.12–4	0.25	1	NA
		*K. pneumoniae*	106	0.12–1	0.25	0.5	0 (0)
		*K. oxytoca*	18	0.25–0.5	0.25	0.5	0 (0)
		*S. marcescens*	16	0.25–4	1	4	NA
		*Enterobacter* spp.	24	0.12–2	0.25	1	0 (0)
Almaghrabi [66]	2014	Carbapenem-R *K. pneumoniae*	50	0.125–1	NA	NA	NA
		Gentamicin-S	30	0.125–1	0.25	0.5	NA
		Gentamicin-R	20	0.125–1	0.5	1	NA
Galani [67]	2012	MDR, XDR, PDR Enterobacterales ^a,b,d^	300	≤0.25–4	1	2	ΝA
		*K. pneumoniae*	241	0.5–4	1	2	0 (0), I: 13 (31.3)
		*E. coli*	33	0.25–2	1	2	0 (0)
		*E. cloacae*	19	0.5–2	0.5	1	0 (0)
		*E. aerogenes*	7	0.5–2	1	1	0 (0)
Pankuch [68]	2011	*P. aeruginosa* ^i^	26	0.5–256	16	64	NA
Landman [72]	2010’	*P. aeruginosa*	679	0.12–>64	8	32	NA
		*A. baumannii*	407	0.12–>64	8	16	NA
Lin [69]	2010	MRSA	47	0.5–8	NA	NA	NA
Livermore [70]	2010	Carbapenem-R Enterobacterales	82	≤0.12–≥256	0.25	≥256	16 (19.5)
Endimiani [71]	2009	MDR *K. pneumoniae* ^d^	102	≤0.125–4	0.5	1	0 (0), I: 2 (2)
		KPC *K. pneumoniae*	25	0.25–1	0.5	1	0 (0)

* Studies are presented in descending chronological order (and alphabetical order within a year). Studies by the same first author, published within the same calendar year are ordered in descending chronological order by month of publication. ** All percentage figures were rounded to one decimal place, except the values < 0.1 that were rounded to two decimal places. Notes: ^a^ PDR: nonsusceptible to all antimicrobial agents in all standard-of-care antimicrobial categories, ^b^ XDR: nonsusceptible to ≥1 antibiotic agent in all but two or fewer relevant antimicrobial classes, ^c^ Isolates from the family of Morganellaceae were included in statistical analysis for resistance calculation, ^d^ MDR: nonsusceptible to ≥1 antimicrobial agent from ≥3 relevant classes of antimicrobials, ^e^ Analyzed isolates expressed at least one 16S rRNA methyltransferase enzyme, ^f^ Resistance data for a set of isolates labeled as challenge isolates were reported. The challenge set isolates were characterized using whole genome sequencing (WGS), and their resistance to aminoglycosides and beta-lactams was determined using Vitek 2, ^g^ Resistance to fluoroquinolones was determined according to CLSI 2019 standards, ^h^ Isolates resistant or intermediate-resistant to ceftazidime and/or ceftriaxone, according to a VITEK-2 instrument and then-current MIC breakpoints, ^i^ Isolates were selected to include resistant phenotypes. Abbreviations: *A. baumannii*, *Acinetobacter baumannii*; AME, aminoglycoside modifying enzyme positive; carbapanemase+, carbapanemase-producing; CLSI, Clinical and Laboratory Standards Institute; *C. freundii*, *Citrobacter freundii*; *C. koseri*, *Citrobacter koseri*; *C. sedlakii*, *Citrobacter sedlakii*; *E. aerogenes*, *Enterobacter aerogenes*; *E. cloacae*, *Enterobacter cloacae*; *E. coli*, *Escherichia coli*; ESBL+, extended-spectrum β-lactamase positive; ESBL−, extended-spectrum β-lactamase negative; I, intermediate; *K. aerogenes*, *Klebsiella aerogenes*; *K. oxytoca*, *Klebsiella oxytoca*; *K. pneumoniae*, *Klebsiella pneumoniae*; KPC, *Klebsiella pneumoniae* carbapenemase; MDR, multi-drug resistant; MIC, minimum inhibitory concentration; MIC_50_, minimum inhibitory concentration 50%; MIC_90_, minimum inhibitory concentration 90%; *M. morganii*, *Morganella morganii*; MRSA, methicillin-resistant *Staphylococcus aureus*; NDM, New Delhi metallo-β-lactamase; NLF, non-lactose fermenting; NS, non-susceptible; OXA-48, oxacillinase-β-lactamase-48; *P. aeruginosa*, *Pseudomonas aeruginosa*; PDR, pandrug resistant; *P. mirabilis*, *Proteus mirabilis*; *P. stuartii*, *Providencia stuartii*; *P. vulgaris*, *Proteus vulgaris*; R, resistant; S, susceptible; *S. aureus*, *Staphylococcus aureus*; *S. epidermidis*, *Staphylococcus epidermidis*; *S. marcescens*, *Serratia marcescens*; VIM, verona integron-encoded metallo-β-lactamase; XDR, extensively drug resistant.

## Data Availability

No new data were created or analyzed in this study. Data sharing is not applicable to this article.

## Supplementary Materials

The following supporting information can be downloaded at: https://www.mdpi.com/article/10.3390/antibiotics15060559/s1, Supplementary File S1: Supplementary table of Risk of bias assessment, Table S1: Risk of Bias Assessment in the included studies; Supplementary File S2: Detailed search strategies applied in each database as of 4 November 2025.

## Abbreviations

AAC	aminoglycoside acetyltransferases
AME	aminoglycoside-modifying enzymes
ANT	aminoglycoside nucleotidyltransferases
APH	aminoglycoside phosphotransferases
CLSI	Clinical and Laboratory Standards Institute
CRE	carbapenem-resistant Enterobacterales
cUTIs	complicated urinary tract infections
ESBLs	extended-spectrum β-lactamases
FDA	Food and Drug Administration
HABA	hydroxy-aminobutyric acid
MDR	multi-drug resistant
MIC	minimum inhibitory concentration
UTIs	urinary tract infections
