# Supplementary material for: Resistance to Plazomicin: An Analysis of the Evidence from In Vitro Antimicrobial Susceptibility Studies
Source: Antibiotics (Basel). 2026 May 30;15(6):559. doi: 10.3390/antibiotics15060559 (PMC13296218; doi:10.3390/antibiotics15060559)
Supplement: Supplementary file 1 [file antibiotics-15-00559-s001.zip › Supplementary File S1. Risk of bias evaluation - revised.pdf]

Supplementary Table S1. Risk of Bias Assessment in the included studies

**Author**	**Year**	**Domains***						**Total**	**Ref. #**
		**1**	**2**	**3**	**4**	**5**	**6**		
Huang	2025	Low	Low	Moderate	High	Low	Low	High	[31]
Zhanel	2025	Low	Low	Moderate	Moderate	Low	Moderate	Moderate	[28]
Dahbouh	2024	Low	Moderate	High	Moderate	Low	Low	High	[34]
Halim	2024	High	Moderate	High	Moderate	High	Low	High	[35]
Markovska	2024	Moderate	Moderate	Moderate	Moderate	Moderate	Low	High	[36]
Markovska	2024’	Low	Moderate	Moderate	Moderate	Low	Low	Moderate	[37]
Sękowska	2024	High	Moderate	Moderate	High	Moderate	Low	High	[38]
Słabisz	2024	Low	Moderate	Low	Moderate	Low	Low	Moderate	[39]
Camargo	2023	Moderate	Low	Moderate	High	Low	Moderate	High	[29]
Cañada-García	2023	Low	Moderate	Moderate	Moderate	Low	Low	Moderate	[46]
Maraki	2023	Moderate	Moderate	Moderate	Moderate	Low	Low	High	[40]
Sader	2023	Low	Low	Moderate	Moderate	Low	Moderate	Moderate	[27]
Teo	2023	Moderate	Moderate	Low	High	Low	Moderate	High	[41]
Zhang	2023	Low	Moderate	High	High	Moderate	Low	High	[42]
Arca-Suárez	2022	Low	Low	Moderate	High	Low	Moderate	High	[44]
Blanchard	2022	Low	Low	Low	Low	Low	High	High	[26]
Gadallah	2022	Moderate	Moderate	High	High	Moderate	Low	High	[43]

Supplementary Table S1. Risk of Bias Assessment in the included studies

Cañada-García	2022	Low	Low	Moderate	Moderate	Low	Low	Moderate	[45]
Gysin	2022	Moderate	High	Moderate	High	Moderate	Moderate	High	[33]
Abd-Elmonsef	2021	Moderate	Moderate	Moderate	High	Moderate	Low	High	[47]
Albano	2021	Moderate	Moderate	Moderate	Moderate	Moderate	Moderate	High	[48]
Essam	2021	Moderate	High	Moderate	High	High	Low	High	[49]
Gysin	2021	Low	Moderate	Moderate	High	Low	Moderate	High	[25]
Huang	2021	Low	Moderate	High	High	Low	Low	High	[50]
Ince	2021	Low	Low	Moderate	Moderate	Moderate	Low	Moderate	[24]
Johnston	2021	Low	Moderate	Moderate	High	Low	Moderate	High	[52]
Johnston	2021’	Moderate	Moderate	Moderate	High	Moderate	Moderate	High	[51]
Maraki	2021	Low	Low	Moderate	Moderate	Low	Low	Moderate	[53]
Clark	2020	Moderate	High	High	High	Low	Low	High	[54]
Fleischmann	2020	Low	Low	Moderate	Moderate	Low	Moderate	Moderate	[55]
Gür	2020	High	Moderate	High	High	Low	Moderate	High	[23]
Jacobs	2020	Moderate	Moderate	High	High	Low	Moderate	High	[56]
Galani	2019	Moderate	Low	Moderate	Moderate	Low	Low	Moderate	[57]
Savov	2019	High	High	High	High	Moderate	Low	High	[58]
Walkty	2019	Low	Low	Moderate	Moderate	Low	Moderate	Moderate	[59]
Zhanel	2019	Low	Low	Moderate	Moderate	Low	Moderate	Moderate	[33]
Castanheira	2018	Moderate	Moderate	High	Moderate	Low	Moderate	Moderate	[21]
Castanheira	2018’	Moderate	Moderate	High	Moderate	Low	Moderate	Moderate	[22]

Supplementary Table S1. Risk of Bias Assessment in the included studies

Thwaites	2018	Moderate	High	High	High	Low	High	High	[60]
Denervaud-Tendon	2017	Moderate	High	High	High	Moderate	High	High	[61]
López-Díaz	2017	High	High	High	High	Moderate	Low	High	[63]
Martins	2017	Moderate	Low	High	High	Low	Low	High	[62]
Zhang	2017	Low	Low	Moderate	High	Low	Low	High	[64]
Rodríguez-Aviala	2015	Moderate	High	Moderate	High	Low	Low	High	[65]
Almaghrabi	2014	Low	Low	Moderate	High	Low	Low	High	[66]
Walkty	2014	Low	Low	Moderate	Moderate	Moderate	High	High	[19]
Galani	2012	Low	Low	Moderate	Moderate	Low	Moderate	Moderate	[67]
Pankuch	2011	Moderate	Moderate	Moderate	High	Low	Moderate	High	[68]
Tenover	2011	Low	Low	Moderate	High	Low	High	High	[30]
Aggen	2010	High	High	High	High	Low	High	High	[18]
Landman	2010	Moderate	Moderate	Low	Moderate	Low	High	High	[20]
Landman	2010'	Moderate	Moderate	Low	Moderate	Low	High	High	[72]
Lin	2010	High	Moderate	Moderate	Moderate	Low	High	High	[69]
Livermore	2010	Low	Moderate	Moderate	Moderate	Low	Low	Moderate	[70]
Endimiani	2009	Moderate	Moderate	High	High	Moderate	Moderate	High	[71]

*Each domain is defined according to the relevant domain definition of the risk-of-bias evaluation tool for *in vitro* antimicrobial susceptibility studies, developed by Falagas et al.: Domain 1 (Methodological bias), Domain 2 (Selection bias), Domain 3 (Preparation bias; including

Supplementary Table S1. Risk of Bias Assessment in the included studies

contamination/cross-contamination bias), Domain 4 (Measurement/Observer bias), Domain 5 (Reporting and Publication bias), Domain 6 (bias related to unreported funding and conflicts of interest) [17].
