# Supplementary material for: Resistance to Plazomicin: An Analysis of the Evidence from In Vitro Antimicrobial Susceptibility Studies
Source: Antibiotics (Basel). 2026 May 30;15(6):559. doi: 10.3390/antibiotics15060559 (PMC13296218; doi:10.3390/antibiotics15060559)
Supplement: Supplementary file 1 [file antibiotics-15-00559-s001.zip › Supplementary File S2. Detailed search strategies.pdf]

Supplementary File S2. Detailed search strategies used in each resource as of 4 November 2025

**Resource**	**Search string**	**Results**
Embase	(plazomicin OR 'achn-490') AND ('antibiotic resistance' OR resistance OR 'non-susceptibility' OR 'non susceptibility' OR nonsusceptibility OR 'reduced susceptibility') AND (mic OR 'minimum inhibitory concentration' OR 'broth microdilution')	215
PubMed	(plazomicin OR "achn-490") AND ("antibiotic resistance" OR resistance OR "non-susceptibility" OR "non susceptibility" OR nonsusceptibility OR "reduced susceptibility") AND (MIC OR "minimum inhibitory concentration" OR "broth microdilution")	48
Scopus	(plazomicin OR "achn-490") AND ("antibiotic resistance" OR resistance OR "non-susceptibility" OR "non susceptibility" OR nonsusceptibility OR "reduced susceptibility") AND (MIC OR "minimum inhibitory concentration" OR "broth microdilution")	796
Web of Science	(plazomicin OR "achn-490") AND ("antibiotic resistance" OR resistance OR "non-susceptibility" OR "non susceptibility" OR nonsusceptibility OR "reduced susceptibility") AND (MIC OR "minimum inhibitory concentration" OR "broth microdilution")	145
